# Diminished plasma levels of common γ-chain cytokines in pulmonary tuberculosis and reversal following treatment

**DOI:** 10.1371/journal.pone.0176495

**Published:** 2017-04-27

**Authors:** Nathella Pavan Kumar, Vaithilingam V. Banurekha, Dina Nair, Subash Babu

**Affiliations:** 1 National Institutes of Health—NIRT— International Center for Excellence in Research, Chennai, India; 2 National Institutes for Research in Tuberculosis, Chennai, India; 3 Laboratory of Parasitic Diseases, NIAID, NIH, Bethesda, Maryland, United States of America; Rutgers Biomedical and Health Sciences, UNITED STATES

## Abstract

**Background:**

The immune response to tuberculosis (TB) is T cell dependent. T cells are the major facilitators of protection and effector functions with CD4^+^ T cells being the most important players, followed by CD8^+^ T cells. The common γ-chain cytokines IL-2, IL-7, IL-15, and IL-21 play a vital role in peripheral T cell growth and survival. However, the role of common γ-chain cytokines in pulmonary TB (PTB) is poorly understood.

**Aim and methods:**

To examine the association of circulating common γ-chain cytokines with TB disease or infection, we examined the systemic levels of IL-2, IL-7, IL-15, and IL-21 in individuals with PTB, latent TB (LTB) or no TB infection (NTB). We also examined the levels of these cytokines in PTB individuals before and after anti-tuberculosis treatment.

**Results:**

Circulating levels of IL-2, IL-7 and IL-21 were significantly diminished in PTB compared to LTB or NTB individuals. Moreover, TB antigen stimulated whole blood also exhibited diminished levels of common γ-chain cytokines in PTB compared to LTB or NTB individuals. The plasma levels of common γ-chain cytokines exhibited no significant association with the severity or extent of TB disease or with bacterial burdens. However, upon standard anti-TB treatment, both the systemic as well as the TB antigen stimulated levels of IL-2, IL-7 and IL-21 were significantly increased in PTB individuals.

**Conclusion:**

Therefore our data demonstrate that diminished levels of common γ-chain cytokines are a common characteristic of PTB and potentially highlight the importance of boosting these responses to improve treatment outcomes.

## Introduction

Tuberculosis (TB) is a significant public health problem. One-third of the world’s population is thought to have latent TB, a condition where individuals are infected by the intracellular bacteria, *Mycobacterium tuberculosis* (Mtb) without active disease but are at risk for reactivation, if their immune system fails [1]. T-cell mediated immune responses are crucial in the regulation of specific host Mtb interactions [2]. It is well known that a central protective immune response against Mtb is cell-mediated immunity [3, 4]. In particular, CD4^+^ T cells are essential for controlling the pathogenesis of Mtb [5, 6]. One of the first steps in the protective immune response against Mtb infection is the expansion of effector T cells following antigen presentation by dendritic cells (DCs) [7, 8]. HIV infection, where in CD4^+^ T cells are depleted, is the most significant risk factor for active TB [9].

Among the family of the common γ-chain cytokines, IL-2, IL-7, IL-15 and IL-21 are the most prominent. These cytokines utilize heterodimeric or trimeric receptors which share the common gamma-chain receptor subunit [10] and play a crucial role in the support of T cell proliferation, survival, and function during immune responses [11–13]. IL-2 is a central cytokine released by activated T cells and exhibits multiple functions, including driving T-cell growth, augmenting NK cytolytic activity and inducing differentiation of regulatory T cells [14]. In addition, IL-2 has also been suggested as a key component in the control and protection against Mtb infection [15]. IL-7 or IL-15 may also contribute to a qualitatively or quantitatively different cytokine milieu during the infection with *M*. *tuberculosis* [16], and signaling by both IL-7 and IL-15 synergizes to promote the generation of memory T cells responses [17]. Previous published data report that macrophages with mycobacteria will lead to effective secretion of IL-7 [18] and IL-15 [19]. IL-21 signaling plays an essential role in T cell responses during Mtb infection by strengthening CD8+ T cell priming, promoting T cell accumulation in the lungs, and boosting T cell cytokine production [20].

Deficiencies in the expression or function of γ-chain cytokines often leads to defects in T cell priming and activation and increases susceptibility to a wide variety of infectious diseases [21]. However, the association of common γ-chain cytokines with TB infection or disease is still not well understood. We hypothesized that active TB would be characterized by diminished levels of one or more of these γ-chain cytokines in comparison to latent TB (LTB) or no TB (NTB) infection. We, therefore, examined the circulating levels of these common γ-chain cytokines in individuals with PTB, LTB and NTB. Our data reveal that circulating levels of IL-2, IL-7 and IL-21 were significantly decreased in PTB compared to LTB or NTB individuals. In addition, TB antigen stimulated levels of the above cytokines were also significantly decreased in PTB compared to LTB. These alterations in cytokine levels were significantly reversed following treatment.

## Materials and methods

### Study population

Patients attending the outpatient clinics of NIRT and community controls were enrolled for this study. This was a prospective case control study and we enrolled consecutive patients and controls. Plasma samples was collected in sodium heparin tubes and stored in -80°C after centrifugation from 66 individuals with active pulmonary TB (PTB), 66 individuals with latent TB (LTB) and 66 individuals with no TB (NTB). The diagnosis of PTB was based on smear and culture positivity. Chest X-rays were used to define cavitary disease as well as unilateral versus bilateral involvement. Smear grades were used to determine bacterial burdens and classified as 1^+^, 2^+^ and 3^+^. At the time of enrollment, all active TB cases had no record of prior TB disease or anti-tuberculosis treatment (ATT). LTB diagnosis was based on Tuberculin skin test and Quantiferon TB-Gold in Tube ELISA positivity, absence of chest radiograph abnormalities or pulmonary symptoms. A positive TST result was defined as an induration at the site of tuberculin inoculation of at least 12mm in diameter to minimize false positivity due to exposure to environmental mycobacteria. NTB individuals were asymptomatic with normal chest X-rays, negative TST (indurations < 5 mm in diameter) and Quantiferon ELISA results. All participants were BCG vaccinated, HIV negative, non-diabetic and had normal body mass index. All participants did not exhibit signs or symptoms of any associated lung or systemic disease. The study groups were similar with regard to age and gender and the baseline characteristics of the study participants are shown in Table 1. Standard anti-TB treatment (ATT) was administered to PTB individuals using the directly observed treatment, short course (DOTS) strategy. At 6 months following ATT initiation, fresh plasma samples were obtained. All PTB individuals were culture negative at the end of ATT. All individuals were examined as part of a study protocol approved by the Internal Ethics Committee of NIRT and written informed consent was obtained from all participants.

**Table 1:** Demographics of study groups.

Study Demographics	Pulmonary TB	Latent TB	Non TB
**No. of subjects recruited**	**66**	**66**	**66**
**Gender (M/F)**	**47/19**	**51/15**	**49/17**
**Median Age (Range)**	**37 (18–64)**	**39 (21–60)**	**41 (19–60)**
**Smear Grade (1**^**+**^ **/2**^**+**^ **/3**^**+**^**)**	**23/18/25**	**Not applicable**	**Not applicable**
**Quantiferon TB Gold ELISA in Tube**	**Not done**	**Positive**	**Negative**
**Tuberculin skin test, mm**	**Not done**	**>12mm**	**<12mm**

### ELISA

Circulating levels of IL-2, IL-7, IL-15 and IL-21 were measured in Quantikine ELISA kit (R&D Systems). The lowest detection limits were as follows: IL-2, 7.813 pg/mL; IL-7, 0.713 pg/mL; IL-15, 1.625 pg/mL; and IL-21, 31.25 pg/mL. The lowest standard value was assigned to the samples that were below the threshold of detection.

### QuantiFERON supernatant ELISA

Whole blood from a subset of individuals with active pulmonary TB (PTB), individuals with latent TB (LTB) and individuals with no TB (NTB) (n = 30 each) was incubated with either no antigen or tuberculosis antigen (ESAT-6, CFP-10, TB 7.7) or mitogen (phorbol ester/ionomycin) for 18 hours, according to the manufacturer’s instructions, using a QuantiFERON In-Tube Gold kit (Qiagen, Valencia, CA). Unstimulated or tuberculosis antigen–stimulated or mitogen—stimulated whole blood supernatants were then used to analyze levels of IL-2, IL-7, IL-15 and IL-21, using Quantikine ELISA kit (R&D Systems). The TB antigen or mitogen stimulated cytokine levels are shown as net cytokines with unstimulated levels subtracted out.

### Statistical analysis

Geometric means (GM) were used for measurements of central tendency. Statistically significant differences between the three groups were analyzed using the Kruskal-Wallis test with Dunn’s multiple comparisons. The Mann-Whitney test was used to compare common γ-chain cytokines concentrations between the individuals with pulmonary TB with unilateral or bilateral lung lesions or cavitary or non-cavitary disease. Linear trend post-test was used to compare angiogenic factor concentrations with smear grades (reflecting bacterial burdens). Wilcoxon signed rank test was used to compare common γ-chain cytokines concentrations before and after ATT. Analyses were performed using Graph-Pad PRISM Version 6.0.

## Results

### Circulating common γ-chain cytokines levels are diminished in PTB and restored following anti-TB treatment

**Fig 1:**
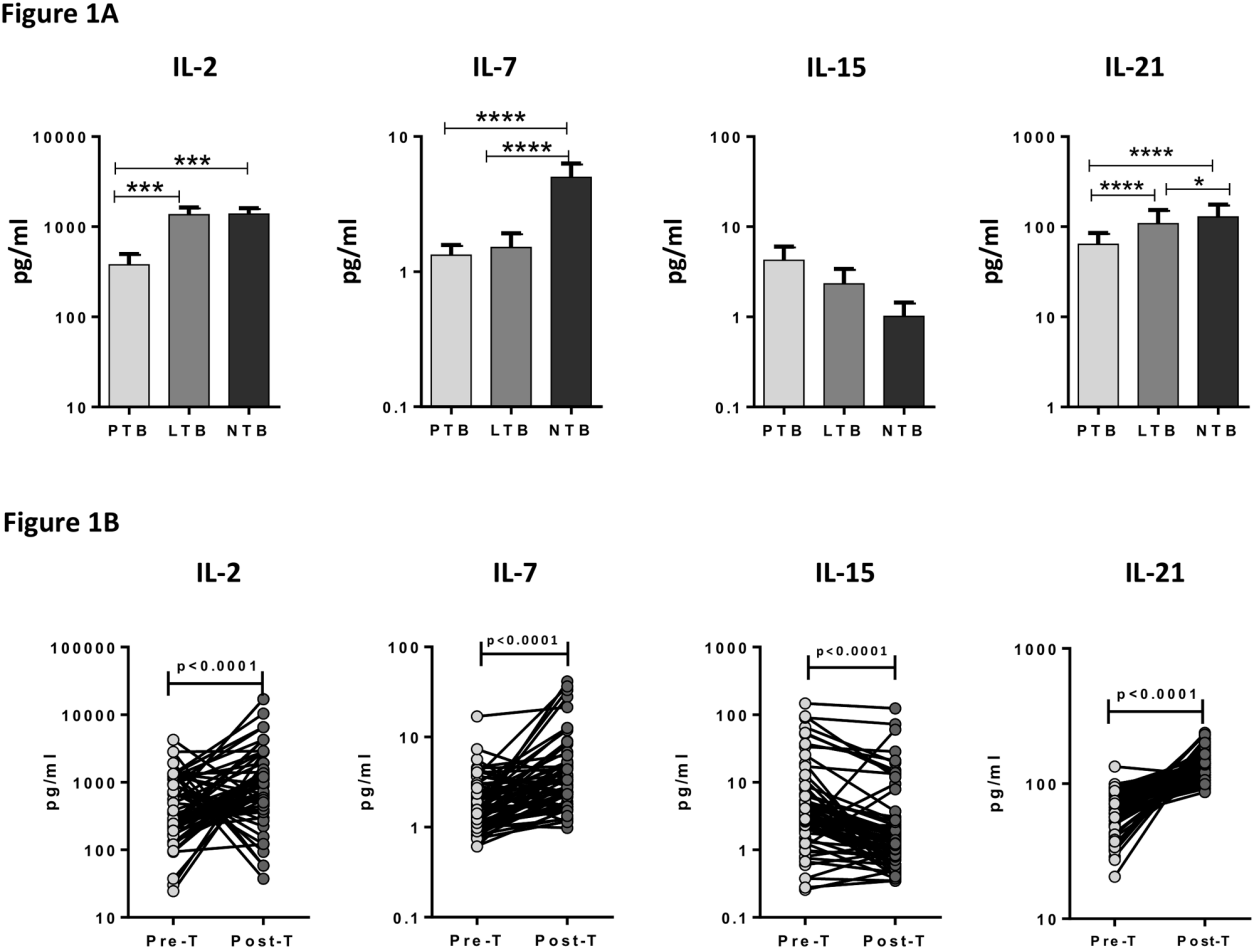
Diminished systemic levels of circulating common γ-chain cytokines in PTB individuals and restoration following anti-TB treatment. (A) The plasma levels of common γ-chain cytokines IL-2, IL-7, IL-15 and IL-2 were measured in PTB (n = 66), LTB (n = 66) and NTB (n = 66) individuals. The data are represented as bar graphs showing geometric means and 95% confidence intervals. P values were calculated using the Kruskal-Wallis test with Dunn's post hoc comparison. (B) The plasma levels of common γ-chain cytokines IL-2, IL-7, IL-15 and IL-2 were measured in PTB individuals before (pre-T) and after (post-T) standard anti-tuberculosis chemotherapy. The data are represented as line graphs with each line representing a single individual. P values were calculated using the Wilcoxon signed rank test.

To determine the systemic levels of common γ-chain cytokines in TB infection and disease, we measured the circulating levels of IL-2, IL-7, IL-15 and IL-21 in PTB, LTB and NTB individuals (Fig 1). As shown in Fig 1A, the systemic levels of IL-2 (GM of 377.9 pg/ml in PTB versus 1364 pg/ml in LTB and 1388 pg/ml in NTB), IL-7 (GM of 1.324 pg/ml in PTB versus 1.507 pg/ml in LTB and 4.990 pg/ml in NTB) and IL-21 (GM of 60.47 pg/ml in PTB versus 99.55 pg/ml in LTB and 120.8 pg/ml in NTB) were significantly decreased in PTB compared to both LTB and NTB individuals. There was no significant difference in the levels of IL-15 amongst the three groups. Thus, PTB is associated with decreased systemic levels of circulating common γ-chain cytokines. In contrast, the levels of IL-2 (GM of 377.9 pg/ml in pre-treatment (Pre-T) versus 895.9 pg/ml in post-treatment (Post-T)), IL-7 (GM of 1.324 pg/ml in Pre-T versus 3.615 pg/ml in Post-T) and IL-21 (GM of 60.47 pg/ml in Pre-T versus 135.1 pg/ml in Post-T) were significantly increased at post- treatment compared to pre-treatment levels in PTB individuals whereas IL-15 (GM of 4.042 pg/ml in Pre-T versus 2.11 pg/ml in Post-T) alone was significantly decreased at post-treatment in PTB (Fig 1B). Thus, successful treatment of active TB results in significantly elevated levels of most circulating common γ-chain cytokines in PTB.

### No association of common γ-chain cytokines levels with extent of disease, disease severity and bacterial burden in PTB

**Fig 2:**
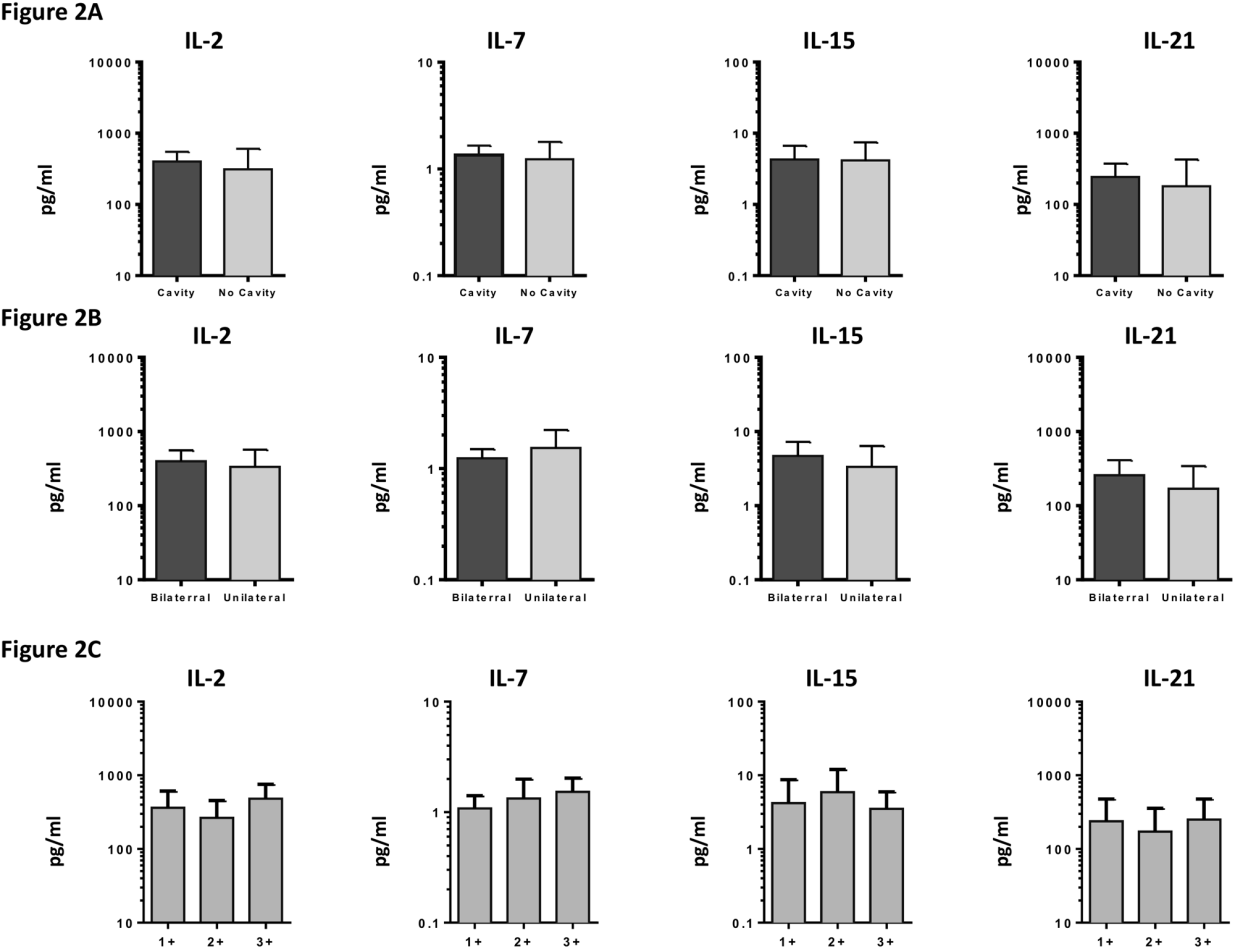
No association of common γ-chain cytokines circulating levels with extent of disease, disease severity or bacterial burdens in PTB individuals. (A) The plasma levels of common γ-chain cytokines IL-2, IL-7, IL-15 and IL-21 in PTB individuals with bilateral versus unilateral disease, reflecting the extent of disease. (B) The plasma levels of common γ-chain cytokines IL-2, IL-7, IL-15 and IL-21 in PTB individuals with cavitary versus non-cavitary disease, reflecting the disease severity. The data are represented as bar graphs showing geometric means and 95% confidence intervals. P values were calculated using the Mann-Whitney test. (C) The relationship between the plasma levels of common γ-chain cytokines IL-2, IL-7, IL-15 and IL-21 and smear grades as estimated by sputum smears was examined in PTB individuals. The data are represented as bar graphs showing geometric means and 95% confidence intervals. P values were calculated using linear trend post-test.

To determine the association between the systemic levels of common γ-chain cytokines and extent of disease and disease severity in PTB, we measured the circulating levels of IL-2, IL-7, IL-15 and IL-21 in PTB individuals with unilateral versus bilateral disease and with cavitary versus non-cavitary disease (Fig 2). As shown in Fig 2A, the systemic levels of IL-2, IL-7, IL-15 and IL-21 exhibited no significant difference in PTB individuals with bilateral disease compared to unilateral disease. Similarly, as shown in Fig 2B, the circulating levels of IL-2, IL-7, IL-15 and IL-21 exhibited no significant difference in PTB individuals with cavitary disease compared to those without cavitary disease. To determine the association between the systemic levels of common γ-chain cytokines and bacterial burden in PTB, we examined the correlation between the circulating levels of IL-2, IL-7, IL-15 and IL-21 in PTB individuals with smear grade classified as 1^+^, 2^+^ and 3^+^ (Fig 2C). As shown, the systemic levels of IL-2, IL-7, IL-15 and IL-21 exhibited no significant correlation with smear grades in PTB individuals.

### TB antigen stimulated levels of common γ-chain cytokines are diminished in PTB and restored following anti-TB treatment

**Fig 3:**
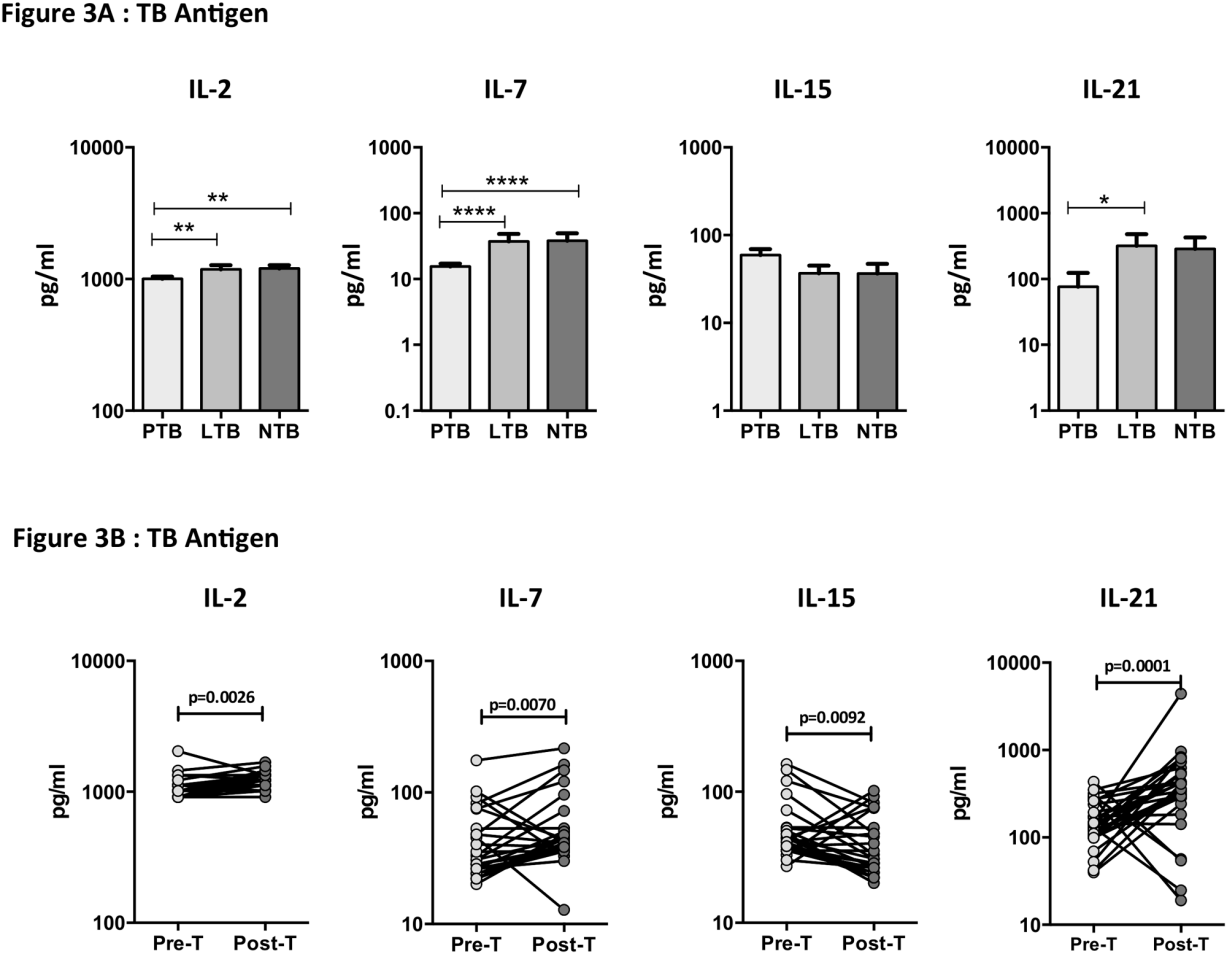
Diminished tuberculosis (TB) antigen–stimulated levels of common γ-chain cytokines in PTB individuals and restoration following anti-TB treatment. (A) TB antigen stimulated levels of common γ-chain cytokines IL-2, IL-7, IL-15 and IL-2 were measured in PTB (n = 30), LTB (n = 30) and NTB (n = 30) individuals. The data are represented as bar graphs showing geometric means and 95% confidence intervals. P values were calculated using the Kruskal-Wallis test with Dunn's post hoc comparison. (B) TB antigen stimulated levels of common γ-chain cytokines IL-2, IL-7, IL-15 and IL-2 were measured in PTB individuals before (pre-T) and after (post-T) standard anti-TB chemotherapy. The data are represented as line graphs with each line representing a single individual. P values were calculated using the Wilcoxon signed rank test.

To determine the influence of TB infection and disease in TB antigen–stimulated levels of common γ-chain cytokines, we measured circulating levels of these cytokines after stimulation with cocktail of tuberculosis antigens (ESAT-6, CFP-10, TB 7.7) or mitogen in a subset (n = 30 each) of PTB, LTB and NTB individuals. As shown in Fig 3A, the TB antigen stimulated levels of IL-2 (GM of 1005 pg/ml in PTB versus 1184 pg/ml in LTB and 1204 pg/ml in NTB), IL-7 (GM of 15.49 pg/ml in PTB versus 37.33 pg/ml in LTB and 38.26 pg/ml in NTB) and IL-21 (GM of 76.15 pg/ml in PTB versus 319.3 pg/ml in LTB and 285.9 pg/ml in NTB), were significantly diminished in PTB compared to both LTB and NTB individuals. There was no significant difference in the TB antigen stimulated levels of IL-15 amongst the three groups. In contrast, tuberculosis antigen–stimulated levels of IL-2 (GM of 1005 pg/ml in Pre-T versus 1197 pg/ml in Post-T), IL-7 (GM of 15.49 pg/ml in Pre-T versus 48.46 pg/ml in Post-T) and IL-21 (GM of 7615 pg/ml in Pre-T versus 319.3 pg/ml in Post-T) were significantly increased at post- treatment compared to pre-treatment levels in PTB individuals (Fig 3B). Thus, successful treatment of active TB results in significantly elevated levels of TB antigen stimulated common γ-chain cytokines in PTB.

**Fig 4:**
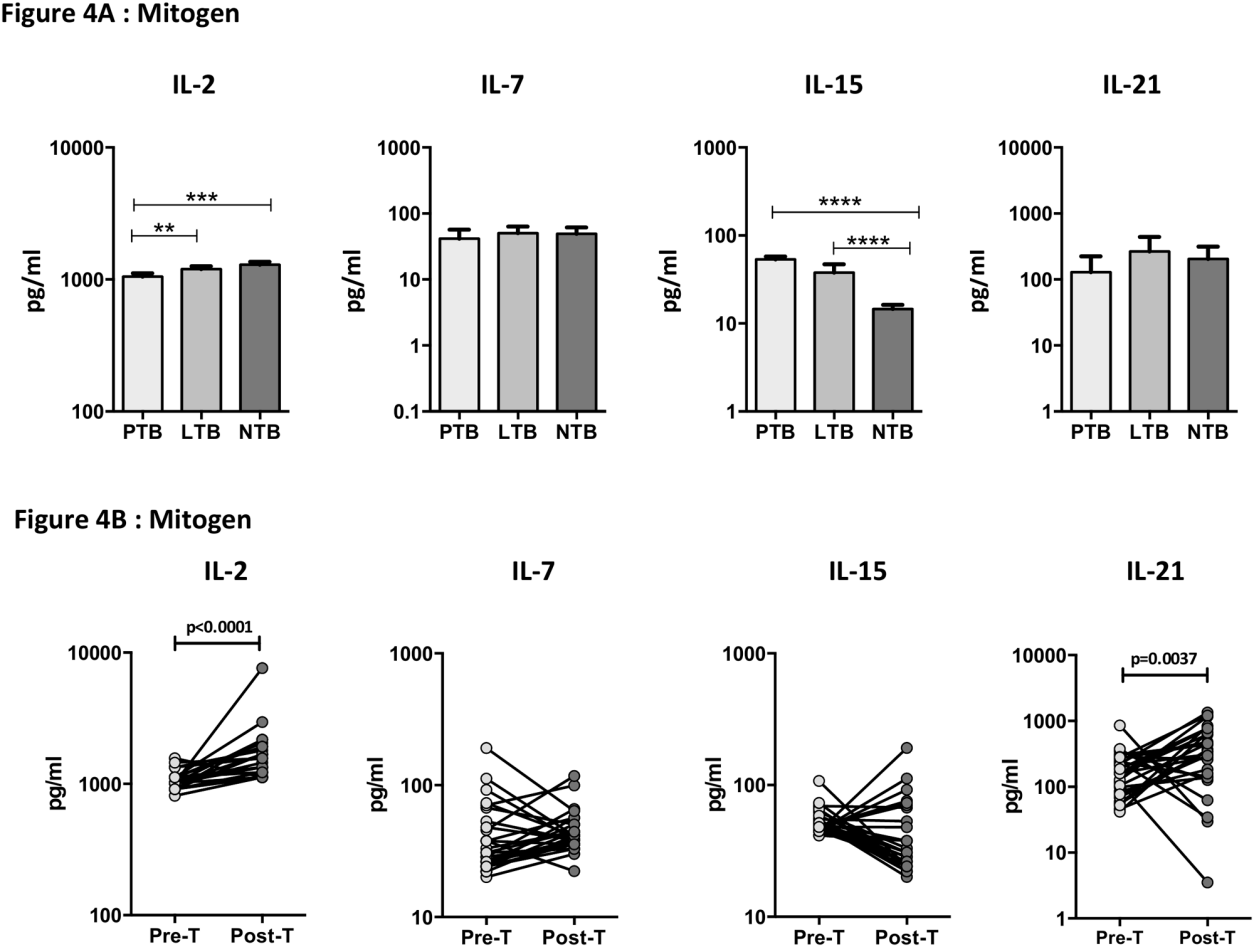
Diminished mitogen stimulated levels of common γ-chain cytokines in PTB individuals and restoration following anti-TB treatment. (A) Mitogen stimulated levels of common γ-chain cytokines IL-2, IL-7, IL-15 and IL-2 were measured in PTB (n = 30), LTB (n = 30) and NTB (n = 30) individuals. The data are represented as bar graphs showing geometric means and 95% confidence intervals. P values were calculated using the Kruskal-Wallis test with Dunn's post hoc comparison. (B) Mitogen stimulated levels of common γ-chain cytokines IL-2, IL-7, IL-15 and IL-2 were measured in PTB individuals before (pre-T) and after (post-T) standard anti-TB chemotherapy. The data are represented as line graphs with each line representing a single individual. P values were calculated using the Wilcoxon signed rank test.

Mitogen stimulation showed diminished levels of IL-2 (GM of 1051 pg/ml in PTB versus 1197 pg/ml in LTB and 1296 pg/ml in NTB) (Fig 4A) but increased levels of IL-15 (GM of 53.32 pg/ml in PTB versus 37.86 pg/ml in LTB and 14.57 pg/ml in NTB) in PTB compared to LTB and NTB. In addition, mitogen stimulated levels of IL-2 (GM of 1051 pg/ml in Pre-T versus 1579 pg/ml in Post-T) and IL-21 (GM of 150.8 pg/ml in Pre-T versus 266.3 pg/ml in Post-T) were significantly elevated post- treatment compared to pre-treatment levels in PTB individuals (Fig 4B).

## Discussion

We have previously explored the role of circulating angiogenic factors in the pathogenesis of TB infection and disease and shown that circulating angiogenic factors serve as biomarkers of disease severity and bacterial burden in pulmonary tuberculosis [22]. Using the same well-characterized group of individuals (PTB, LTB and NTB), we explore the role of common γ-chain cytokines IL-2, IL-7, IL-15, and IL-21 in the pathogenesis of TB disease in this study. The common γ-chain cytokines play a central role in peripheral T cell expansion, function, and survival [23], are important growth factors for T cells [24], and therefore are presently being directed *in-vivo* as immunotherapy in certain diseases [25]. The common γ-chain is essential for the function of at least four cytokines including IL-2, IL-7, IL-15 and IL-21, which jointly modulate lymphocyte growth and control a broad range of activities that will shape the innate and acquired immune responses [26]. However, the role of common γ-chain cytokines in TB infection and disease has not been explored in detail. Our study on the homeostatic (or steady state) levels of common γ-chain cytokines reveals several major features.

Cytokines of adaptive immune systems orchestrate the immune response to tuberculosis infection, with Th1 cytokines having been associated in protection against tuberculosis disease [26, 27]. First IL-2 promotes T-cell replication and is crucial for cellular immunity and granuloma formation in TB infection [28, 29]. IL-2 is predominantly expressed by T cells, mainly the CD4^+^ Th1 subsets and also by activated CD8^+^ T cells and dendritic cells (DCs). Diverse immunological studies have shown that IL-2 serum levels has been significantly diminished in active TB disease compare to latent or healthy controls at baseline or upon TB antigen stimulation [30–32]. Our data clearly expand on these reports and confirm that IL-2 exhibits decreased levels in PTB compared to LTB or NTB at homeostasis or upon TB antigen or mitogen stimulation and levels were significantly restored after successful completion of anti-TB treatment. Although, the levels of IL-2 in our study were quite high, they are consistent with our previous observations in PTB individuals in the same area [33]. The IL-2 levels in the healthy controls are comparable to previously published levels in the same cohort [34]. However, the IL-2 levels in our study are different from the IL-2 levels observed in other studies [35]. Thus, PTB individuals appear to have both an intrinsic defect as well as an antigen-responsive defect in the optimal production of IL-2 in TB disease. IL-2 is known to exhibit therapeutic usefulness in different disease states and exogenous delivery of IL-2 promote development of effector T cells to control chronic infection in animal models [36]. Studies have also reported that in the Mtb infected macaque model, IL-2 treatment concurrently expanded regulatory T cells and T effector cells and conferred resistance to severe TB inflammation. [37]. Similarly, IL-2 is also used in clinical trial to deliver significant benefit in the case of certain cancers and infectious diseases, including HIV [38]. Therefore, IL-2 as host- adjunct therapy could conceivably benefit immunity to TB disease.

IL-7 signaling supports the survival and homeostatic proliferation of naive and memory CD8^+^ T cells [39, 40]. Studies have also reported that IL-7 has been involved in regulating naive T cell homeostasis [41]. Previously published data reports that IL-7 is elevated in patients with tuberculosis compared to latent TB or healthy controls [42, 43], but in contrast other studies report that IL-7 response to stimulation with TB antigen displayed diminished cytokine levels compared to healthy controls [44]. Similar to the latter results, our data also show that IL-7 exhibits decreased levels in PTB compared to LTB or NTB at homeostasis or upon TB antigen stimulation and levels were significantly restored after successful completion of anti-TB treatment. IL-7 is also known for its therapeutic usefulness in different disease states including HIV, wherein, IL-7 treatment improved the percentage of IFNγ secreting Gag-specific CD4^+^ T cells in HIV patients [45]. In addition, it has been reported that IL-7 would be most useful during the acute phase of HIV infection [46]. IL-7 concentrations in previously published data from HIV uninfected healthy individuals are almost similar to our non-TB healthy controls [47]. Studies from animal experiments report administration of IL-7 and IL-15, with BCG resulted in an improved CD4 and CD8 T cell memory response. Mice injected with BCG supplemented with IL-7 and IL-15 displayed improved T cell proliferation, Th1 cytokine production, and an improved Mtb specific memory T cells as proven by a significant improvement in protection against *M*. *tuberculosis*. [48]. Our data on the defective induction of IL-7 in PTB individuals would also suggest that IL-7 is another target for host-directed therapy in active TB disease.

IL-21, a potent immunomodulatory cytokine, has role on both innate and adaptive immune responses [49, 50]. T helper subsets are able to produce IL-21, however Th17 cells appear to be the most abundant producers of IL-21 [51]. IL-21 can induce the activation, proliferation and differentiation of T cells and also enhances the proliferation and differentiation of the macrophage and granulocyte lineages [52]. IL-21 also displays a similar profile to IL-2 and IL-7 in our study. IL-21 also showed decreased levels in PTB compared to LTB or NTB at homeostasis or upon TB antigen stimulation and levels were significantly restored after successful completion of anti-TB treatment. This further confirms our previous finding on decreased plasma levels of IL-21 in PTB using a different group of patients [53, 54]. Similar to our findings, studies from HIV infected patients have shown impaired IL-21 during acute HIV infection when compared to uninfected controls [55] and that this impairment was partially rescued when ART was initiated [55]. Previous published IL-21 levels from the healthy donors were also similar to our IL-21 levels from our non-TB healthy controls cohort [56]. In mouse models, it has been reported that IL-21 signaling has a key role in promoting the protective capacity of T cells. IL-21 signaling enhances host resistance to *M*. *tuberculosis* [20]. Other immunological studies have also reported that HIV specific CD4^+^ T cells from uninfected control showed greater number of IL-21 producing T cells compared to viremic patients [57].

IL-15 a cytokine that links the innate and adaptive immune systems has multiple effects [58] that eventually leads to immune-regulatory cross-talk between natural and specific immune cells [59]. Various studies have sought to determine the circulating levels of IL-15 levels and most studies reveal an enhanced IL-15 response in active TB disease compared to latent TB during baseline or upon TB antigen stimulation [30, 60]. In our study, IL-15 displays contrasting profiles to IL-2, IL-7 and IL-21 with tendency to increase in PTB (though not statistically significant) compared to LTB or NTB. In addition, we do observe significantly increased levels of IL-15 upon mitogen stimulation and this was significantly diminished after successful completion of anti-TB treatment. Studies from HIV infected patients report that higher serum IL-15 levels have been correlated with better control in chronic infected HIV patients [61]. However, our studies have failed to reveal a major association of PTB with diminution of IL-15 levels.

Another interesting feature of our study is the fact that although IL-2, IL-7 and IL-21 exhibited major differences between PTB individuals on the one hand and the control groups on the other, no significant correlation was observed with levels of these cytokines and disease severity, extent of disease and bacterial burdens. But, the post-treatment levels of IL-2, IL-7 and IL-21 in PTB individuals are not significantly different from the baseline levels in LTB or NTB. While this does not completely negate a role for these cytokines in the pathogenesis of these disease features, they do suggest that the T cell promoting common γ-chain cytokines are more associated perhaps with susceptibility or resistance and less with disease pathogenesis.

In summary, the current study delivers evidence that the common γ-chain cytokines IL-2, IL-7 and IL-21 are associated with the differential cytokine responses seen in PTB compared to LTB or NTB individuals. Since this was an exploratory study with small sample size, we are unable to draw any inferences on cause and effect. Being follow-up study in design, our study also clearly describes the progression of the cytokine profile with treatment. Finally our data also suggest common γ-chain cytokines could serve as attractive candidates to be used in immunotherapy as adjunct measures to standard ATT to combat active TB, especially the drug resistant forms.
